# Supplementary material for: The herbaceous landlord: integrating the effects of symbiont consortia within a single host
Source: PeerJ. 2015 Nov 3;3:e1379. doi: 10.7717/peerj.1379 (PMC4636405; doi:10.7717/peerj.1379)

The herbaceous landlord: Integrating the effects of symbiont consortia within a single host

Supplemental Figures

Roo Vandegrift*[1]
Bitty A. Roy[1]
Laurel Pfeifer-Meister[1]
Bart R. Johnson[2]
Scott D. Bridgham[1]

[1] Institute of Ecology and Evolution, University of Oregon, Eugene, OR, USA
[2] Department of Landscape Architecture, University of Oregon, Eugene, OR, USA

* Corresponding Author:
Roo Vandegrift
335 Pacific Hall,
5289 University of Oregon,
Eugene, OR 97403-5289
540.588.4101
awv@uoregon.edu

Supplemental figures:

Figure S1: Percent root length colonized by AMF and DSEs were correlated positively (Adjusted $R^2 = 0.107$, $F_{1, 153} = 19.51$, $P < 0.001$), indicating facilitation rather than competition. See also Fig. 4 for SEM results.

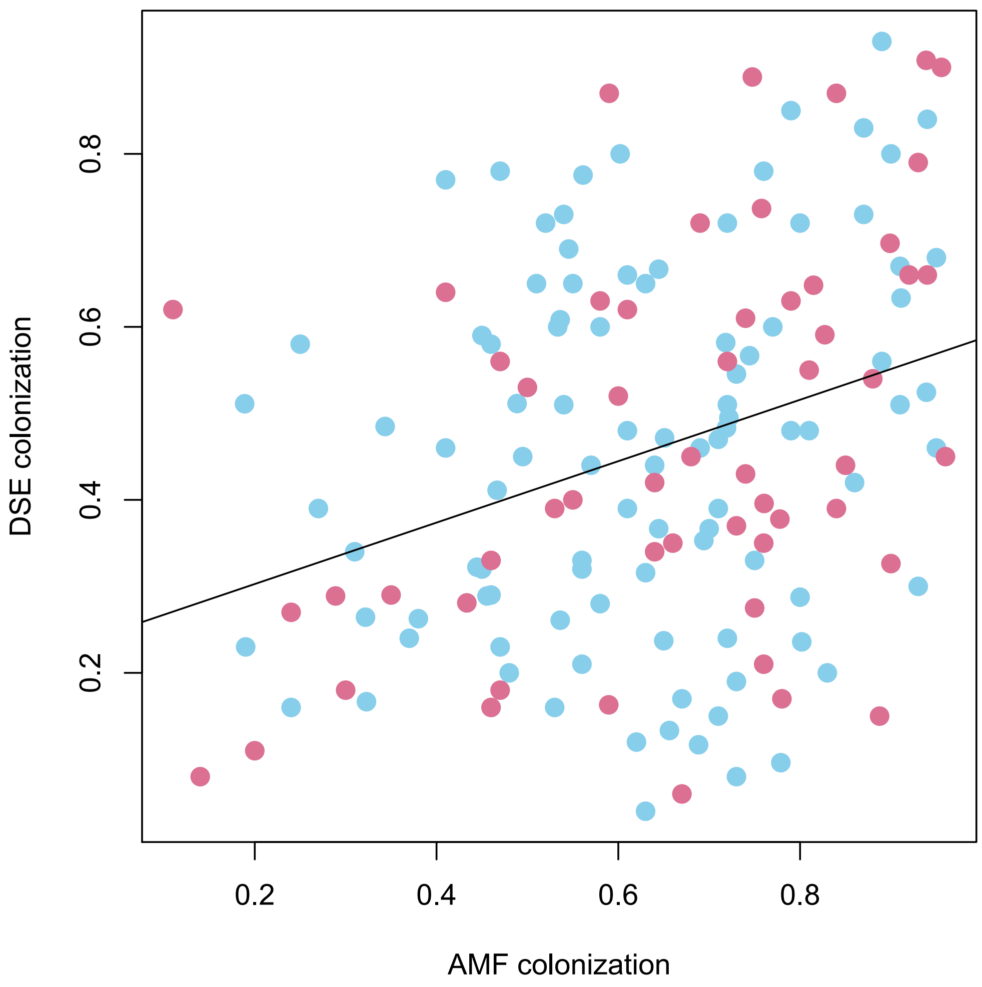

Figure S2: There was a negative correlation between DSE root length colonized and plant biomass, but only in the absence of *Epichloë* infection (E+ Adjusted $R^2 = 0.029$, $F_{1,54} = 2.644$, $P = 0.110$; E− $R^2 = 0.053$, $F_{1,97} = 6.437$, $P = 0.013$). See also Fig. 4 for SEM results.

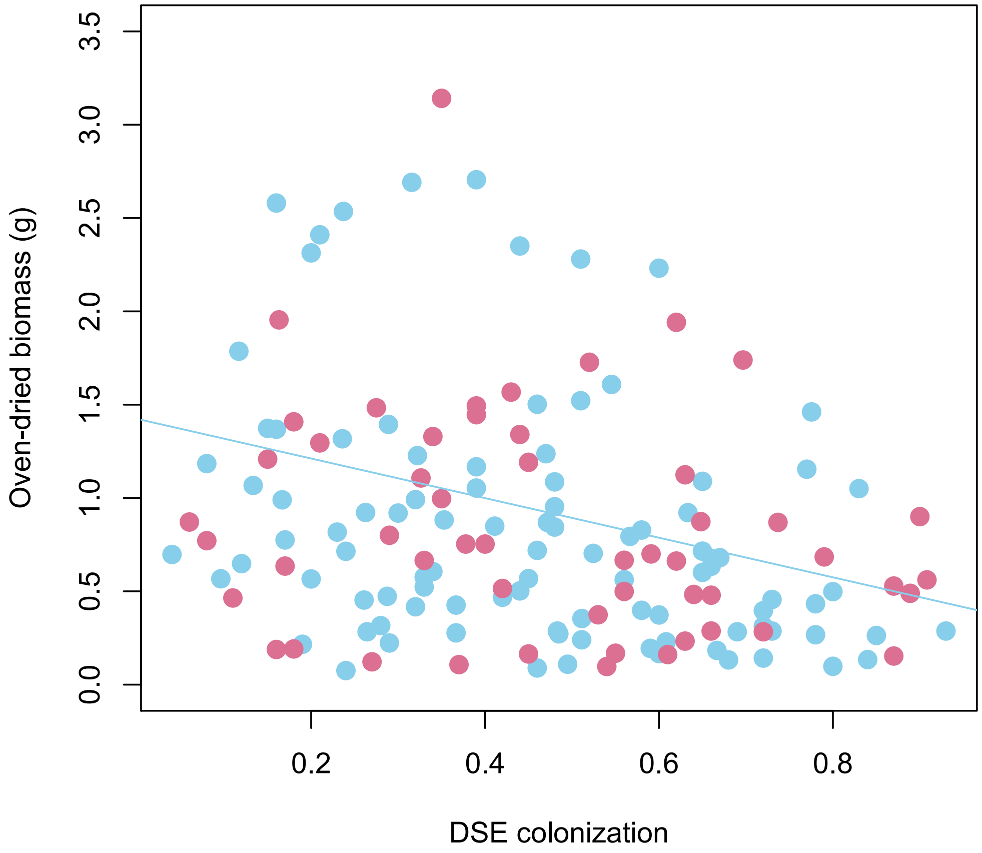

Figure S3: DSE colonization decreased as more water was available to plants (Adjusted R^2 = 0.107, $F_{1, 153}$ = 19.5, $P < 0.001$). See also Fig. 4 for SEM results.

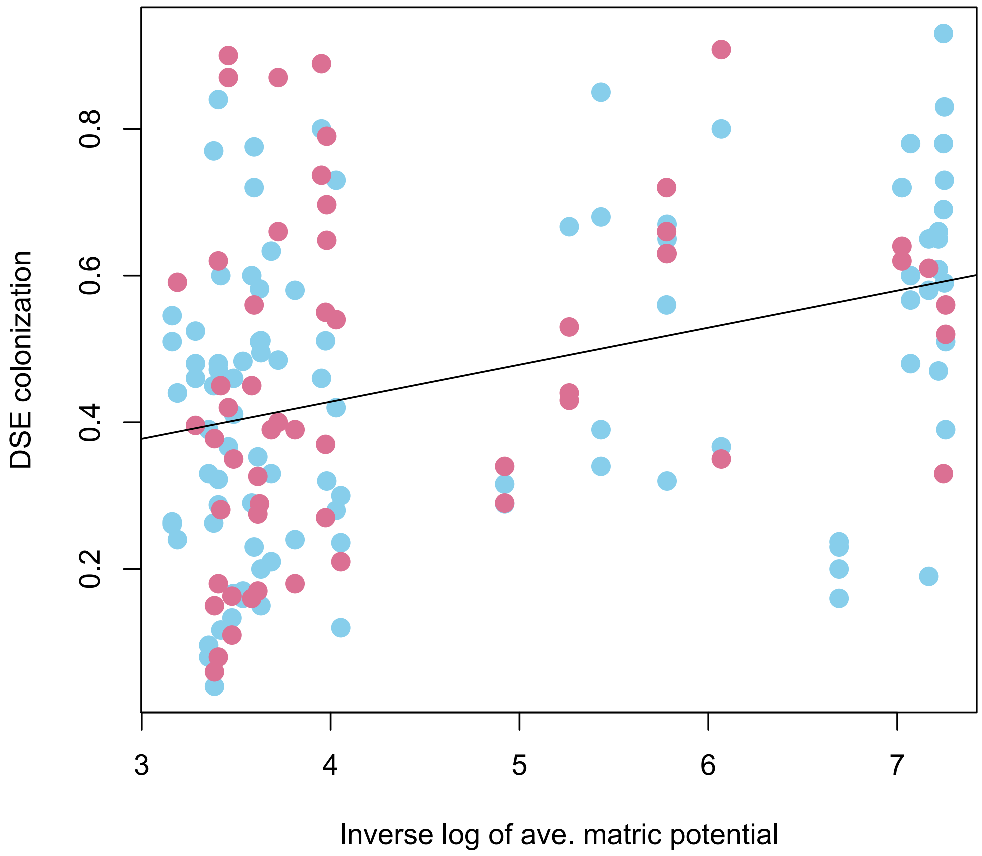

Figure S4-S9: Neither AMF colonization nor proportion of plants hosting *Epichloë* varied significantly with measured edaphic conditions (soil moisture, soil temperature, soil N:P ratios). See also Fig. 4 for SEM results.

Figure S4

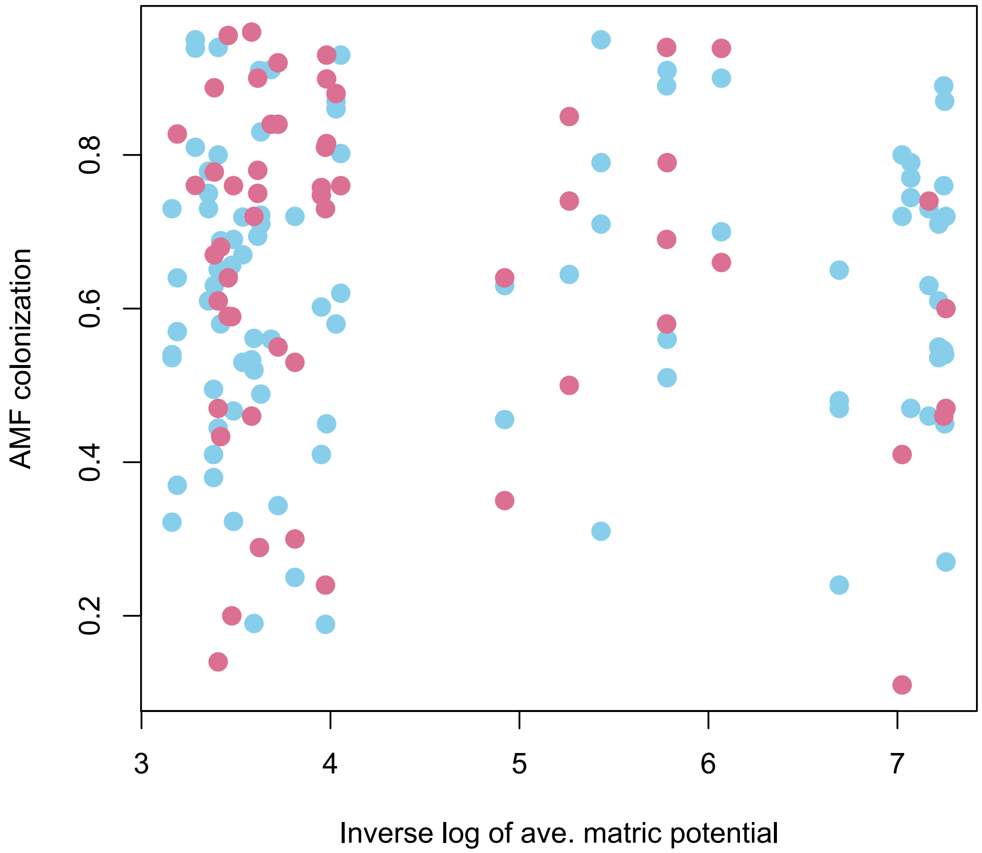

Figure S5

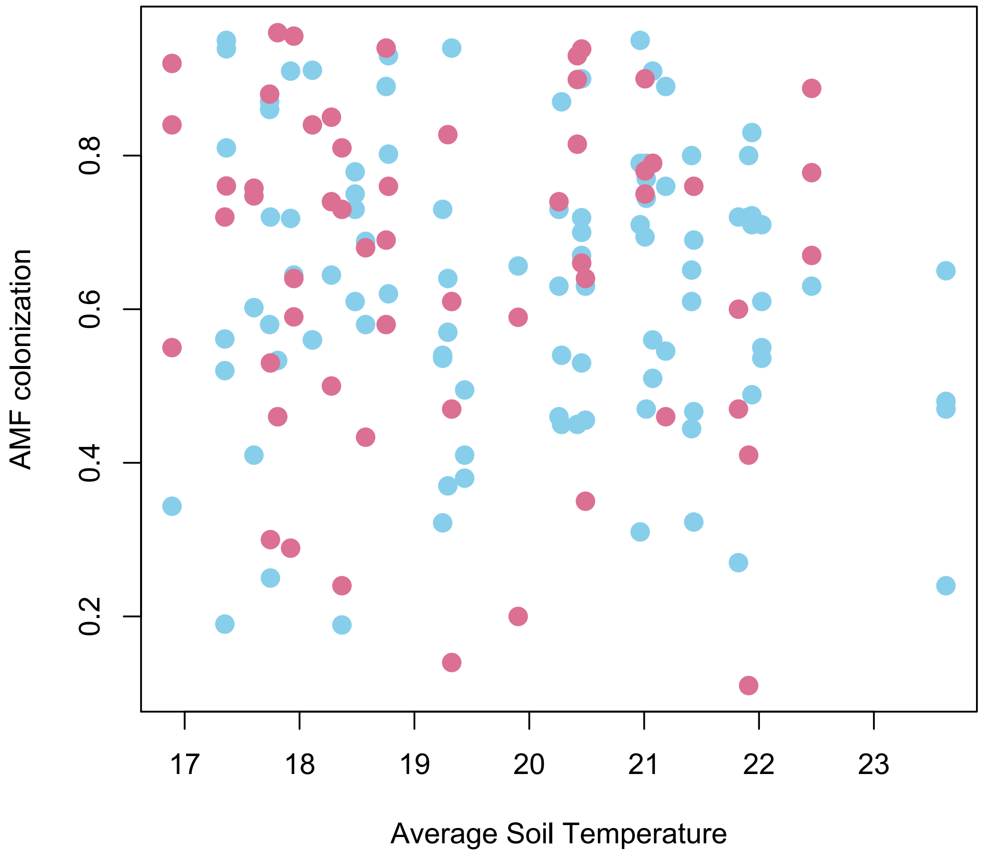
AMF colonization
0.2
0.4
0.6
0.8
17
18
19
20
21
22
23
Average Soil Temperature

Figure S6

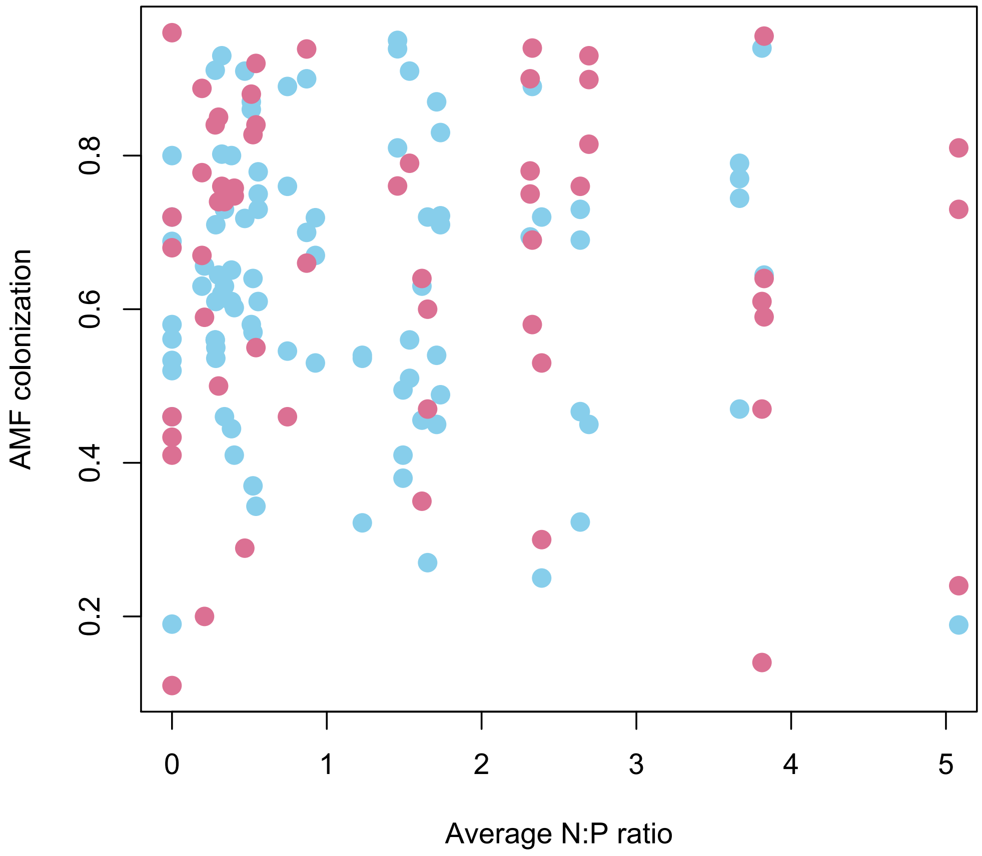

Figure S7

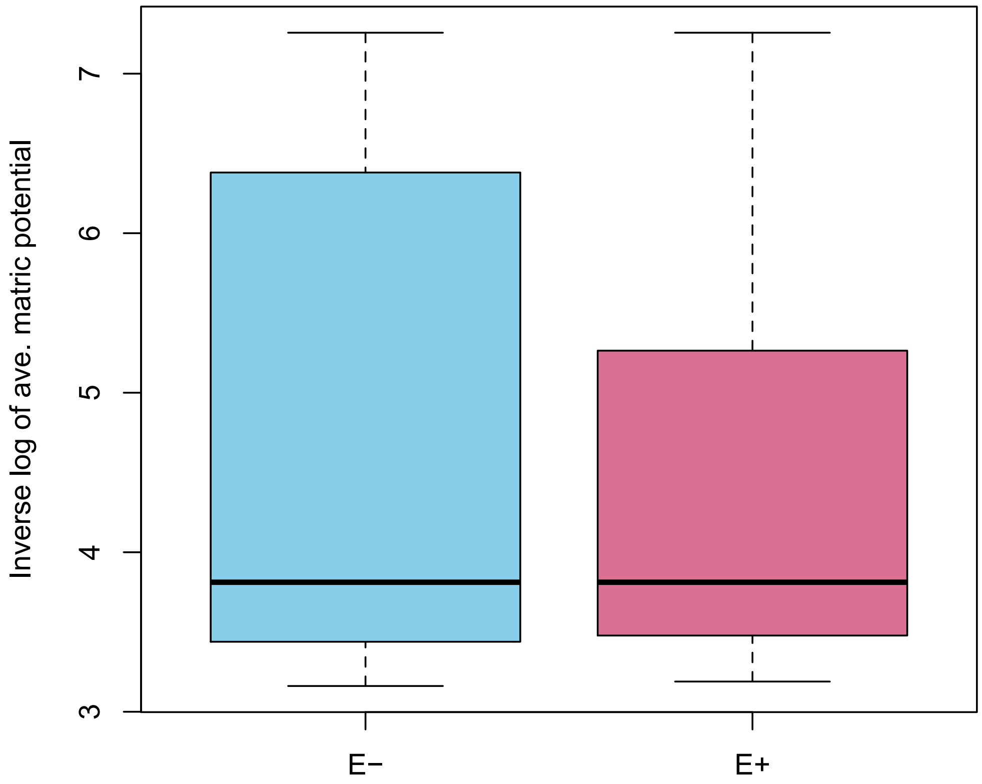

Figure S8

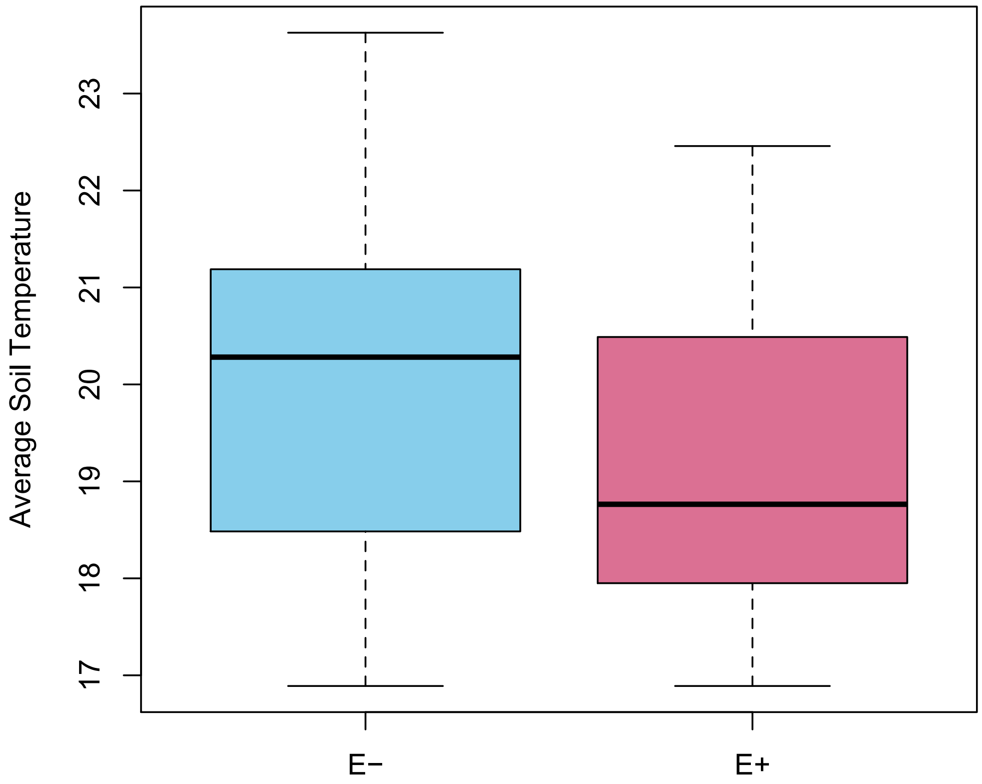

Figure S9

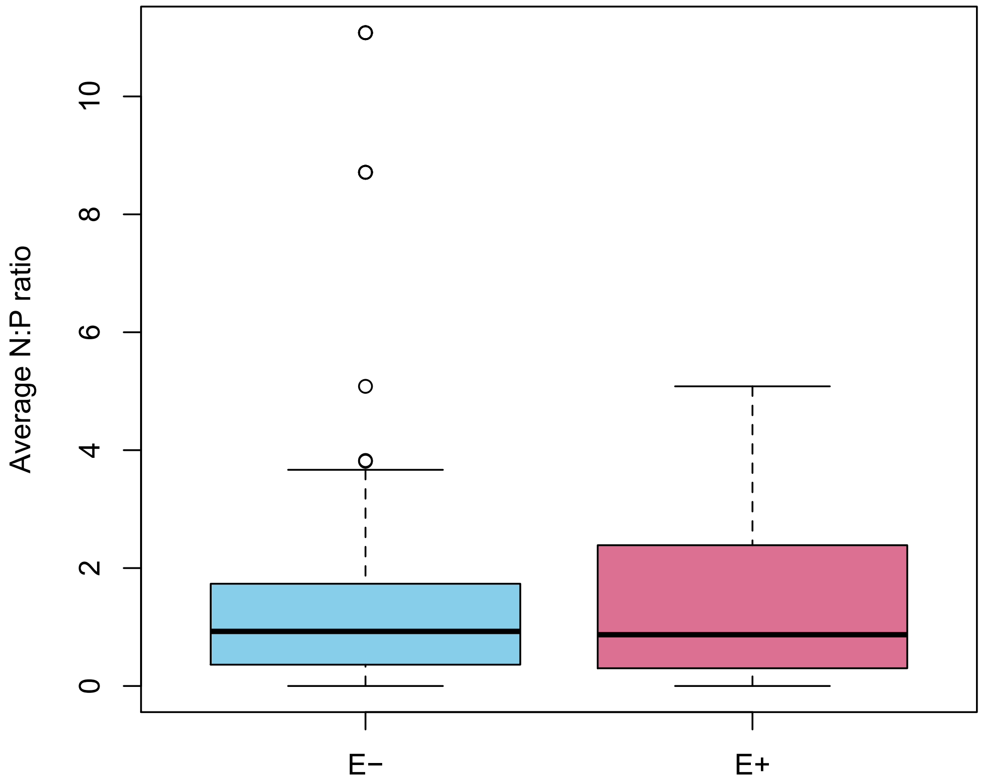

Figure S10: There was effect of *Epichloë* presence on plant fitness as measured by aboveground biomass.

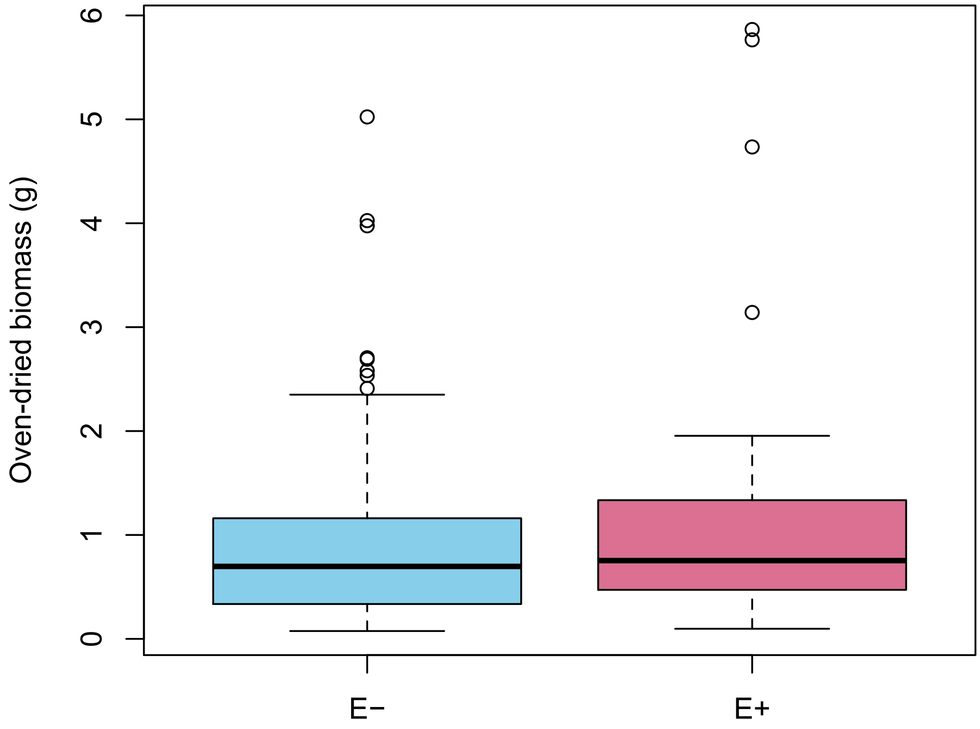

Figure S11: There was effect of AMF colonization on plant fitness as measured by aboveground biomass.

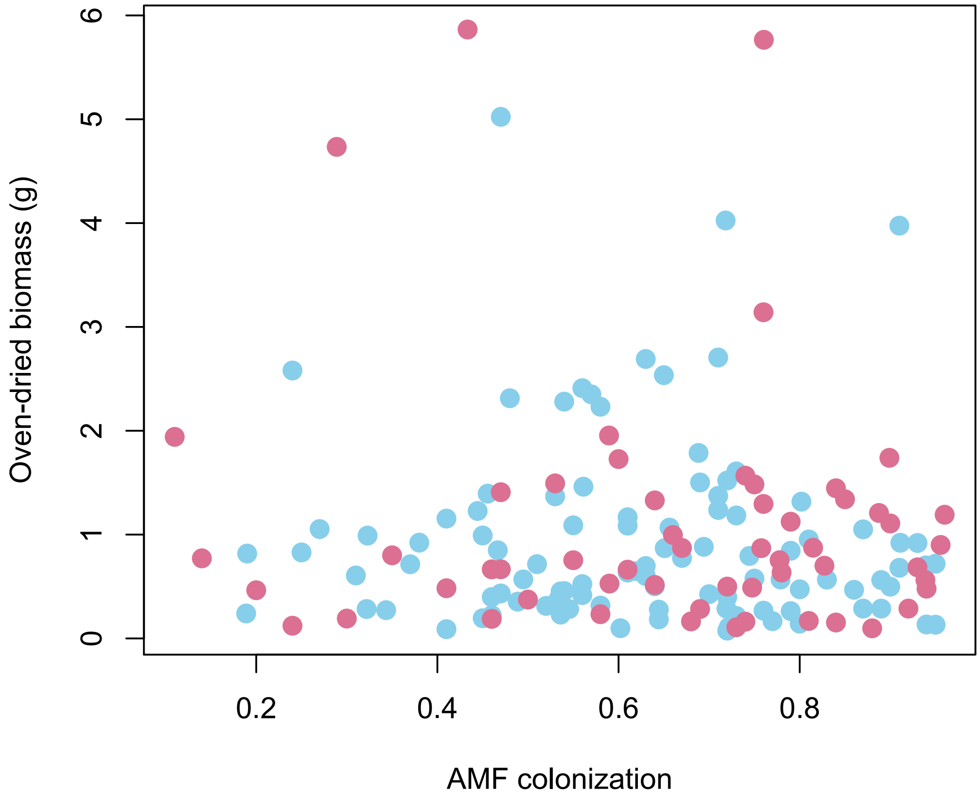

Figure S12: Overall SEMs for *Bromus hordeaceus*, with different models for those plants without *Epichloë* endophytes (A: E−, blue), and those with *Epichloë* endophytes (B: E+, red). Model fit was good for both models, though the low sample size for the E+ may potentially pose issues with interpretation (A: χ^2 = 2.588, P = 0.274; CFI = 1.000; RMSEA = 0.000; n= 83 | B: χ^2 = 4.601, P = 0.100; CFI = 1.000; RMSEA = 0.000; n = 19). The numbers above the arrows are the standardized path coefficients. Non-significant (P > 0.05) path coefficients are not shown. Numbers in the boxes are total explained variance (R^2) of each variable.

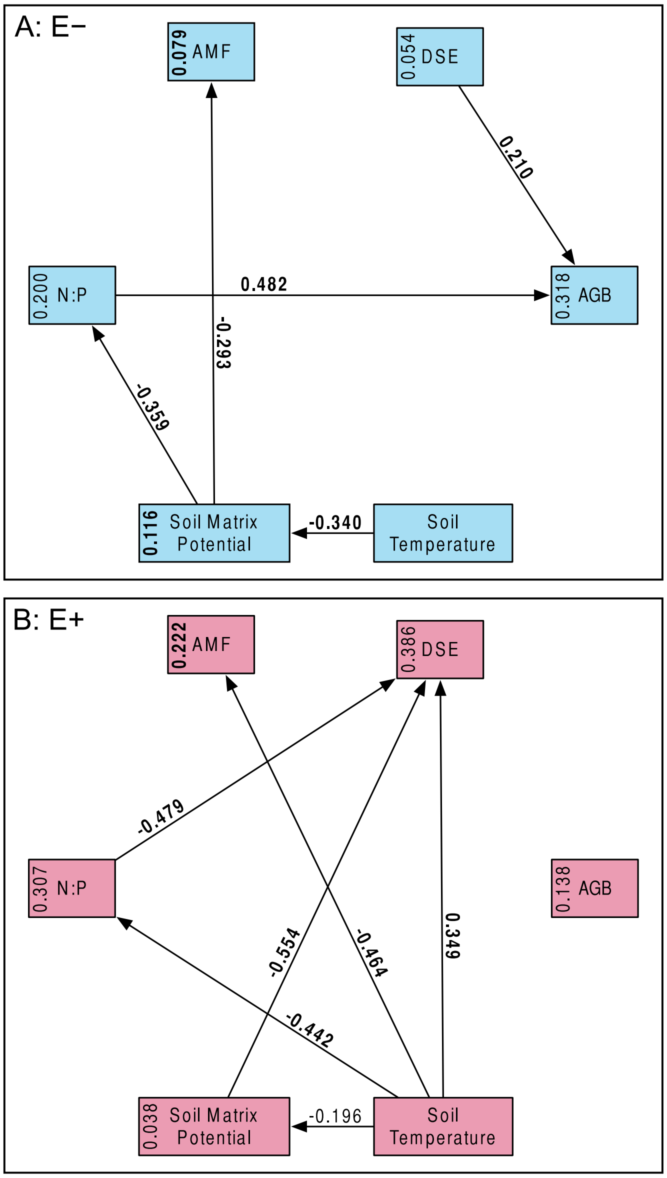

Supplement: Supplemental Information 1 [file peerj-03-1379-s001.pdf]
